# A Practical Treatise on Uterine Hæmorrhage

**Published:** 1832-10-01

**Authors:** 


### A Practical Treatise on Uterine Hæmorrhage, in connexion with Pregnancy and Parturition. By *John T. Ingleby*, Member of the Royal College of Surgeons in London, one of the Surgeons to the General Dispensary, Surgeon to the Magdalen Asylum, and Lecturer on Midwifery at the School of Medicine in Birmingham. One Plate, 8vo. pp. 276. London, 1832.

*(Continued from page* 214 *of this Volume.)*

Uterine hæmorrhage is particularly apt to occur after quick labours. To obviate this Dr. Clarke recommends, in addition to Osborne's plan of retarding the passage of the head, that the contractions of the uterus may be assisted by the other hand placed on the abdomen for the purpose. This practice was recommended by Dr. Gooch, and is considered by the author as a measure calculated materially to effect the object in view. In proof of this a case is adduced, in which a mechanical impediment had the effect of delaying the expulsive efforts, and of preventing hæmorrhage, which on former occasions, when the labours had been very quick, had taken place immediately after the birth of the child in the same patient, to such a degree as to expose her life to imminent danger. In such alarming instances as these the surgeon should invariably, after the birth of the child, promote the contraction of the uterus by external pressure, and take care to avoid all extracting force with the funis. By adopting these precautions the hour-glass-contraction, which is one of the greatest misfortunes attending hæmorrhage from the uterus, and a frequent concomitant of it, may be avoided.

The vomiting, which accompanies *laceration of the uterus*, generally distinguished by its sudden and violent attack, appears to the author to be in great measure dependent on the effusion of blood consequent to the injury. He had an opportunity of examining the bodies of three persons who died from this accident. In two of them blood was found extravasated profusely within the pelvis and abdomen, and vomiting had supervened immediately after the rupture. The third patient had no vomiting, and the quantity of blood, discovered on dissection, did not exceed an ounce.

Rupture of the uterus has been generally considered a fatal accident. A case of this kind occurred in our practice some years ago, in which the rupture was so complete, that it extended through the peritoneal coat, and enabled us to pass the hand amidst the other contents of the abdomen. After experiencing all the usual alarming symptoms peculiar to this accident and severe peritonitis, the woman at length perfectly recovered.

On the subject of *vomiting* the following question occurs :—

"When the vomiting which attends early pregnancy continues, in persons who have previously enjoyed good health, through the middle and latter months, increasing in severity and accompanied by a dangerous prostration of strength, I wish to ask, whether, in the failure of all ordinary resources, and particularly in the absence of any organic disease, the membranes should be ruptured with a view of inducing premature labour ?" P. 41.

In his lectures, published in the Lancet, Dr. Blundel, as a last resource, proposes, but does not appear to have adopted this expedient. Conquest observes that it is now and then essential to the safety of the patient. In the author's practice one patient expired before the completion of the ninth month from exhaustion; in a second, death speedily followed delivery; a third was decidedly relieved by the spontaneous rupture of the membranes; a fourth connected with abdominal encysted dropsy, which brought on a fatal termination, was relieved by the induction of premature labour. In our own practice several instances of excessive prostration of strength and exsanguine aspect have been produced by extreme and continued vomiting in the advanced stages of pregnancy. They have all been relieved by the recumbent posture, attention to the bowels, and the regular daily administration of the citrate of potash in a state of effervescence. In these cases the mode and period of exhibiting aperients is of the utmost importance. Should any obvious necessity arise in such cases to puncture the membranes, we should have no hesitation to adopt such an expedient; all the common resources having been attempted in vain. We should first be disposed to try the effect of hydrocyanic acid, which in the practice of Dr. Elliotson of London, and Mr. Garner of Nottingham, seems to have acted with specific influence in removing this symptom. Of its curative properties in the treatment of pyrosis we can speak with the fullest confidence.* The author has not thought it necessary to mention the effect of local bleeding or stimulation in the epigastric region; but from the high opinion we have formed of his extensive information, we have no doubt he has availed himself of such expedients.

Since we have seen some of the functions of the cerebrum, the cerebellum, and spinal medulla suspended, and other extraordinary morbid sympathies developed by utero-gestation, and removed by parturition; we should be inclined to recommend the puncture of the membranes in all alarming diseases, evidently associated with and induced by the gravid uterus; and, when the operation is conducted with care towards the termination of pregnancy, it will generally be followed by safety to parent and child.

For the relief of *ascites, occurring during pregnancy,* and proceeding from serous inflammation in the free surface of the peritoneum, Mr. Ingleby asserts, in opposition to the advice of Dr. Blundel, that paracentesis of the abdomen is infinitely preferable to the evacuation of the liquor amnii. When bleedings, bandages, diuretics, &c. have no effect, Dr. Blundel directs us to rupture the membranes: as he observes that death, under these circumstances, has taken place from hydrothorax.† The author next details the particulars of the case of dropsy, recorded by Mr. Langstaff in the Medico-Chirurgical Transactions, in which paracentesis abdominis was had recourse to with complete success.

*Sickness* in the earlier months of pregnancy seems to be almost essential to the process: it is at least generally present; and a case came under the observation of our friend, Mr. J. M. Coley of Bridgnorth, in which it was found necessary to excite it daily, in order to obviate abortion, which had repeatedly taken place apparently from its absence alone.

From exhaustion, mental agitation, or the collapse consequent on the sudden expulsion of the fœtus, *syncope* sometimes takes place, especially when the application of the bandage has been neglected. In this way sudden death may also occur. The means of relief consist in external pressure on the abdomen, the horizontal position, stimulants, and cool fresh air. The attack is liable to supervene under circumstances, in which an atonic state of the uterus is not present, and the mind appears to have been antecedently depressed by some unhappy forebodings. An instance of this kind is recorded, in which death was the consequence; and we could add several others to the melancholy catalogue.

When fainting is occasioned entirely by profuse hæmorrhage, the use of stimu-

* For further information respecting the exhibition of prussic acid we refer our readers to Lancet, Vol. XX. p. 202, and p. 650.—*R.*

† Lancet, Vol. XV. p. 420.

lants requires the nicest management; as it is one of [illegible] effectual means which Nature provides for the suspension of the discharge. This rem[illegible] may however be carried too far, and, if not averted by art, may prove fatal. By a judicious use of stimulants, of which subcarbonate of ammonia is one of the safest, at the proper crisis, life may often be restored, after it has apparently been entirely lost. Our friend, Mr. J. M. Coley, to whom we have before alluded, thus succeeded in reviving a female, who had been left by her surgeon as a dead person, and absolutely laid out by her attendants. When not fatal, long-continued syncope from excessive loss of blood is generally followed by disordered function in the heart and brain. The influence exerted by hæmorrhage on the heart is of the most frequent occurrence; as dyspnœa, violent palpitations, hurried circulation, and a peculiar state of pulse, indicating a want of tone or contractile property in the arteries. This condition of the heart and arteries is best relieved by subcarbonate of iron in combination with small doses of digitalis occasionally intermitted.

Amongst the cerebral affections resulting from violent hæmorrhage may be mentioned epilepsy, paralysis, or some disturbance of the mental functions.

Much dispute has existed respecting the properties of *opium*, and its use in uterine hæmorrhage. The author is of opinion that, when administered to a patient exhausted by this cause, it acts in a large dose as a cordial, and appears to him to possess advantages over the common spirituous stimuli. In support of this opinion he adduces the authority of Burns, Gooch, and Hamilton. The condition in which it is found most beneficial, is that in which great restlessness and irritability are present; and the dose in which the author prescribes it, is from one to two drachms of the tincture, and from two to five grains of the powder. When deglutition is suspended, the opiate may be injected per anum. As large a dose as ℥ss. of the tincture has been successfully administered, without any unpleasant consequences. It may be necessary to repeat the medicine at distant intervals, until all danger of collapse should disappear. Among the unpleasant secondary, or sedative effects of the opium, retention of urine is not uncommon; although it appears to have escaped the author's vigilant attention. The indiscriminate use of narcotics, after every labour, is very prudently objected to by him.

As an auxiliary means of restraining uterine hæmorrhage, napkins moistened with cold water should be applied to the pubic region and the loins. The author lays much stress upon the frequency and the suddenness of the application, which may be aided by pouring the water from a height above the patient. As soon as we have accomplished our object, the remedy should be set aside, and the state of collapse, if present, cautiously removed, by warmth conveyed to the epigastrium and extremities. When the external use of cold water has not succeeded, the author has witnessed the most active contractions produced by its injection into the cavity of the uterus. In protracted hæmorrhages he observes that cold solutions of alum and the injection of cold water into the intestines, according to Dr. Hamilton's directions, have been most beneficial.

The author seems to be a convert to the belief of the existence of *muscular fibres in the uterus*, and to the pressure produced by their contraction he attributes almost entirely the suspension of hæmorrhage. This process, he thinks, is much promoted by the excess of lymph prevailing in the blood during gestation, which appears to him to be designed by Nature for the purpose of closing the mouths of the vessels and preventing that oozing, which would otherwise continue after contraction. Whether the walls of the uterus be built with muscular fibres or with a substance sui generis, it is very obvious that they possess the power of contracting the capacity of the organ, and that this is the principal instrument by which the diameter of the bleeding vessels is reduced; in short by which the powerful effects of pressure are accomplished in a situation, where, without its co-operation, external agents would be unavailing.

The author next enter[illegible]minous account of the *structure and physiology of the* [illegible], and concludes by adopting the view taken by Dr. Blundel of the [illegible]ure of the communication between the parental and fœtal circulation.

"On the whole I cordially enter into the view propounded by Dr. Blundel, that the communication is by exceedingly minute orifices or tubes, capable of transmitting the more subtle (subtile) parts of the blood, for instance the serum and the coagulable lymph, to the exclusion of the red particles. This partial independence of life is doubtless designed to answer some wise purpose of the animal economy—perhaps to obviate impressions, both physical and mental, calculated either to impair the regular growth of the body, or to destroy life. Sir E. Home's alleged discovery of nerves in the placenta appears to be mere imagination." 75.

The specific effect of the *ergot, or spur or horn of rye,* which appears to be a fungus, to which that grain is liable, is next noticed. The author's experience does not seem to have afforded him any proof of its possessing the power of originating uterine action, which has been attributed to it by some writers, particularly Dr. Neale.

"The ergot often induces violent uterine action. The degree of violence may be inferred from the fact, that a spasmodic disease is described by Tissot, as resulting from the use of bread, containing ergotted rye. I have generally found its effects uncertain; and, exclusive of its frequent adulteration, as the ergot is a morbid change of the grain of the rye, its properties vary with the variation of the season. This uncertainty cannot be altogether ascribed to the inefficacy of the diseased grain; since the same specimen has produced a material effect upon one individual, yet not the slightest effect upon another, even when exhibited in the same doses and form. In many instances it either produces no effect at all, or is too transient in its operation to promote the expulsion of the child. In such cases, the dose may be renewed on the action subsiding, and by rousing the dormant energies when the pains are either defective or totally suspended, it occasionally exerts a very beneficial influence.

It sometimes causes *extreme* suffering. Dewees indeed states that the ergot does not occasion great pain. My experience upon this point is the reverse of this. So distressing did its operation prove in three instances, that in subsequent labours the patients resolutely refused it. Its full action generally arises speedily after its administration, and is manifested, first, by the womb on external examination becoming tense and firm, and secondly, by the supervention of pains at very short intervals. To this however an exception is to be made. The ergot every now and then defeats itself, in consequence of the action induced, though not strong, being without any *perfect* intermission; the frequency of the contractions seem to prevent their attaining that degree of force which is requisite for the expulsion of the child. That the uterine action is really to be attributed to the operation of this medicine, may be inferred from the complete change which occurs in the character of the pains very soon after its first exhibition. Even under regular and strong paroxysms, the patient, in some instances, does not in the interval obtain freedom from pain, probably from the tonic contraction not entirely ceasing. It is observed by Dr. Cusack, in the 5th Vol. of the Dublin Hospital Reports, that in three instances, where it was employed in half drachm doses, substance as well as infusion being administered, symptoms of an apoplectic nature supervened, such as a diminution in the frequency of the pulse, the beats averaging from fifteen to thirty in a minute, stupor, epistaxis, &c. Though the pulse is said to become under its administration rather slower than ordinary, I do not understand that the symytoms referred to by Dr. Cusack have in other instances been found to occur. Professor Burns, under the impression that morphia forms one of its constituent properties, regards the principle of its action as analagous to that of opium: to this, however, an exception may fairly be taken, since opium will directly arrest its action. Moreover it is proved by the analysis of Vauquelin, and by the more recent analysis of Maas, that morphia is not a component part of the ergot of rye." 80.

The experience of Dr. Cusack tends to corroborate the statement of Tissot, who asserts that, in 1596, an epidemic disease prevailed in Hesse, which the physicians attributed to bread made with horned rye. Some of the patients were attacked with *epilepsy*, which was generally fatal; others with insanity, which left them stupid the rest of their lives. The author proceeds to point out the necessity of observing the dimensions of the pelvis and os uteri and the condition of the uterus and parts opposing its action, before the exhibition of the medicine is decided upon. In a first labour he considers it inadmissible, also in a state of plethora, spasm, undue mechanical resistance, excessive uterine sensibility, and when turning is likely to be required, except in cases of hæmorrhage.

"Again, when the placenta is retained either by spasm or morbid adhesion, the ergot will decidedly be improper. It is applicable to states of inertia *only*. To insure its effects as far as possible, it is very material to administer it in a fresh state, and when the stomach is empty. It may be exhibited in the form of tincture, infusion, or powder; or it may be used as a lavement. In the process of decoction, the active property of the ergot in some measure escapes. Milk added to preparations of the ergot is said to prove an antidote to its violent effects; but if the ergot be proper, what necessity is there for an antidote? especially since its action, when too violent, may be immediately controled by opium. Perhaps it might be administered with advantage in placenta presentation, directly before the hand is conveyed into the uterus, in order to induce that efficient and permanent contraction, which is essential to the patient's security. In ordinary turning, a high degree of contraction is a serious obstacle; but as these peculiar cases of delivery are often accompanied by a dangerous depression of the vital energies, a powerful uterine contraction, that shall be exerted immediately the turning is completed, is most earnestly to be desired. I submit this as a mere conjecture, not having as yet formed an experimental acquaintance with the subject. Perhaps in any case of turning, or instrumental delivery, attended with a deficient uterine action, it may be prudent to exhibit the ergot, in order that the system may be influenced, if possible, before the operation is undertaken. The risk, as well as the great difficulty of extracting a child, when unaided by the proper contraction of the organ, (unless the vital powers be exceedingly depressed, and the relaxation universal), can be appreciated by the experienced only." 83.

In proof of the violent effects produced by the ergot in some instances, and of the necessity of observing circumspection in its employment, the author observes—

"I am informed by a surgeon of extensive practice in this neighbourhood, that to his knowledge several instances of ruptured uteri have really occurred under its violent action." 81.

As far as our own experience goes, we find that the action of the uterine fibres produced by this medicine is different to that which is natural: in the former case the fibres are drawn into rigid, irregular, permanent rugæ, accompanied with corresponding pain of the same continued and spasmodic character; in the latter, the action is intermitting, more of an expulsive kind, the uterine tumour less rugous, and the pains alternating with intervals of ease. This peculiar property of exciting in the generality of instances a specific contraction in the walls of the uterus had led to the use of the ergot, with a view of preventing hæmorrhage after the birth of the child. The author's experience does not enable him to speak decidedly in recommendation of its employment with this intention. We are, however, disposed to think more favourably of it in such cases; at the same time we perfectly agree with the following conclusions:

"As to the practical deductions from the foregoing remarks, I would observe, that additional experience is wanting to confirm the utility of the ergot in uterine hæmorrhage: implicit reliance cannot be placed upon it. At present, therefore, it must in this respect be regarded as of doubtful efficacy. The evidence in its favor, as being calculated to promote uterine contraction under every possible circumstance, though multiplied in extent, establishes no definite principle on which the practitioner may confidently repose. It should be regarded only as an auxiliary, for although it is sometimes efficient in producing the full tonic effect, and thus arresting flooding, it frequently fails, either wholly or partially." 85.

It sometimes happens that no *secretion of milk* takes place after labour. The sympathy existing between the natural and diseased condition of the uterus and breasts induced us to administer, in a case of this kind, an *infusion of rye* on the sixth day after delivery, at which time there was no indication of the lactiferous process commencing. In less than twenty-four hours a copious lacteous secretion followed. Whether this event, which was looked for with great anxiety, was promoted by the rye or not, we must leave the experience of others to determine. It was however a singular and striking circumstance.

The subject of *abortion* is introduced by a scientific explanation of the manner in which the ovum accomplishes its connexion with the uterus. For a particular detail of this process we must refer to the work itself. After reviewing the different theories, which have been advanced, to explain the cause of abortion, the author adverts to the changes which follow conception. Some are of opinion that miscarriage is owing to a deficiency, others to a redundancy of blood, or increased vascular excitement in the maternal system. The author inclines to the latter, and believes that this misfortune is mostly preceded by an increased activity in the circulation, either general or partial. For the treatment of such patients, as are supposed to labour under a defective supply of blood, the cold bath, nutritious diet, gentle exercise, attention to the bowels, and tonics, are recommended. Those, whose abortions are supposed to be occasioned by a generally or partially excited circulation, or to plethora, should submit to the repeated abstraction of moderate quantities of blood during the first four months, beginning just before the termination of the first conception. Care should be taken to avoid syncope. Collateral aid may be afforded by simple spare diet, regular state of the bowels, pure air, perfect rest and cool temperature. The recumbent position should be enforced, as much as possible, and all violent exercise must be avoided. The attachment between the placenta and uterus is so slight in some females, that a ride on horseback, or in a carriage, or a long walk, has been sufficient to lacerate their connexion. When there has existed a tendency to retroversion of the uterus, which generally manifests itself at the same period, when abortion takes place, the author has found opiate suppositories possess singular efficacy. Opium indeed will often prevent the occurrence of premature labour as well as abortion, when administered for the relief of uterine pains, before hæmorrhage has been established. In these cases it will be found advantageous to combine venæsection and long-continued horizontal rest with the opiate. The author also enjoins the rigid observance of *divortium a toro mariti* from the first to the fifth month. Two cases are appended in illustration of the success of this treatment.

It seems to be an instinctive property of the uterus to enlarge in proportion with the growth of the fœtus, and when the latter is deprived of life or ceases to increase, a disposition to expel its contents commences, and sooner or later expulsive efforts follow. This principle is found to operate even in extra-uterine fœtation; and, as soon as the ovum and its contents have arrived at maturity, the symptoms of parturition begin. Hence, when the deadly poison of vicious intercourse has extinguished the spark of embryo-life, the uterus ceases to enlarge, and rejects the blighted offspring. Thus we believe the perfect development of the uterine inmate is sometimes prevented by primitive imperfection in the ovum or in the delicate process of germination. When the uterus has acquired the habit of acting abortively from the fatal influence of the venereal poison on the germ, we have uniformly found this vicious habit removed by a prudent employment of mercury, which we have prescribed during gestation and afterwards. The author, however, advises us to avoid exciting mercurial action, if possible, until after delivery; it might promote the effect it is intended to prevent.

The author next enumerates the symptoms of abortion, and very prudently urges a careful examination of the discharges. The placenta is sometimes expelled with the cyst and fœtus, at others, it is retained several days or weeks. When it has been long retained, there is danger of slight but troublesome prolapsus uteri following, unless the patient is subjected to the recumbent position.

Among the signs denoting the existence of pregnancy the following remarks occur respecting mediate auscultation.

"On applying the ear to the abdomen of a pregnant woman, with or without the aid of a stethoscope, a whizzing or hissing murmur is distinctly heard, synchronizing with the mother's pulse, and termed the 'placental soufflet' or 'bruit placentaise.' This sound is supposed to depend upon the transmission of blood through the arteries of the uterus at the site of placental attachment, and possibly through the arterial tubes and cells of its maternal portion also; and may be perceived, though somewhat modified, even after the fœtal circulation has ceased. The double pulsation of the fœtal heart, which may also be heard, is an infallible evidence of a fœtus in utero. To this the soufflet must be considered subordinate. But when the fœtus has perished at a very early period of utero-gestation, before the action of the heart can be clearly ascertained, the soufflet alone is available. It is stated that the sound of the fœtal heart has been detected as early as the tenth week; but, antecedent to the fifth month, deception on this point is very likely to arise. To the opinions here advanced respecting the soufflet as a test of pregnancy, an exception has been taken. It is said that the soufflet may be heard even in ordinary enlargements of the uterine vessels, and independently altogether of conception." 101.

After a portion of the placenta has been detached, it is supposed that no reunion ever takes place; and this view coincides with our own observations. When the fœtus has been some time dead, and a necessity for a continuance of the increased determination of blood, previously existing in the uterine circulation, has ceased, the enlargement of the vessels, as well as the orgasm producing it, subsides.

"Under this state of congestion, the placental vessels are rendered either wholly impervious, or still admit of a very imperfect and diminished circulation; and it has been rationally conjectured, that the blood taken up by anastomosing channels, instead of passing through the arteries of the cellular part of the placenta, is directly conveyed to the uterine veins, and from them conducted back to the maternal system; a supposition which accounts for the occasional absence of hæmorrhage in certain cases where the fœtus has been some time dead, as in such instances the uterine circulation has had time to establish itself." 102.

The length of time, during which a dead fœtus may be retained in utero, is uncertain. The usual period is from one to three weeks. Dr. Blundel says—

"When the ovum dies in the earlier months, it may be retained till the close of pregnancy."

In support of this assertion Mr. Ingleby adduces several remarkable cases, in one of which we find an ovum was retained three months after its twin had been expelled.

Mental emotions have in all ages been arranged among the causes of abortions, and the author is confident that a severe fright may instantly destroy fœtal existence without the supervention of hæmorrhage, until some time afterwards. The Father of British Midwifery, some of whose notions were very crude, was also convinced of the same fact.

"Item, aborcement may happen by over-much stirring of the body in labouring, dancing, or leaping, or by some fall or thrust against some wall, or beating, or by some *sodaine anger, feare, dread, sorrow,* or *sodaine and unlooked-for joy.*"*

The *morbid condition of the placenta* here attracts the notice of the author. It sometimes assumes a grape-appearance.

"In form, these bodies are usually oblong, the exterior resembling the decidua uteri, the interior not unlike a bunch of grapes; each eminence being covered with a very thin membrane, and having a dusky red fluid interposed, the fluid in each portion freely communicating. Masses of hydatids, or small vesicles, are often appended both externally and internally. In structure, the lobes vary, being soft, homogeneous, and of a pale red colour, in some; while in others, they appear like coagula enclosed in thin membranous septa. Many of these growths resemble the firmer polypi, or fleshy tubercles, having an imperfect internal membrane. A few of them, except being longer in form, and heavier, differ little from the healthy ovum, having a single cavity only, containing a turbid or bloody fluid. The vestige of a very small fœtus may sometimes be discovered within the ovum, in a rude and undigested shape, attached by a minute filament, its size being in an inverse ratio to that of the placenta. The fœtus must, however, maintain its vitality, though it ceases to grow with the mass which encloses it; and this is the more remarkable, as in one case, which occurred under my observation, the funis was extremely short, and not thicker than a good sized bristle. But, though fœtal or rather embryotic existence may not be extinct, the mass may, to all intents and purposes, be considered a foreign body. The observation made by Leake and Hunter, that a real conception may be dissolved, and pass off in a fluid form, leaving the placenta adhering and increasing in size, accords with experience. Gooch, however, speaks of the ovum being produced without the fœtus. In other abortions of this kind, the mass comes away more consolidated in appearance, and having no definite form. A few of them in shape resemble the cavity in the uterus." 108.

The placenta is sometimes converted almost entirely into a congeries of hydatids, which, like the *volvoces globatores,* are impregnated with smaller ones. We met with a specimen of that kind, expelled at the third month.

After exposing the absurdity of M. Duges and others in attempting to establish any practical or useful distinction between arterial and venous hæmorrhage in the early months of gestation, the author observes that, before the beginning of the fourth month, abortion rarely proves fatal; notwithstanding the quantity of blood discharged is sometimes prodigious and the effects severe. Hence the amount of the flooding is not so much dependant on the existing calibre of the vessels as on the excited or inflammatory condition of the arterial system, evinced

* The Byrth of Mankinde. By Thomas Raynalde, Physitian. London. To be sold by John Morret, at the 2 Tuns in Little Britaine. Black Letter, pp. 136.—*R.*

by the excess of coagulable lymph in the blood, by thirst, dryness of the skin, and increased activity in the heart and arteries; circumstances which prevail more in the earlier than the latter stages of utero-gestation. It is always desirable to limit the effusion of blood, and to afford every possible chance of safety to the fœtus.

"But when, in addition to the hæmorrhage being considerable in quantity, frequent in return, and protracted in duration, general and regular contractions of the womb are excited, and the os internum has undergone a partial dilatation, there can be no chance of the process of gestation being resumed; preventive measures will be useless; and our great object will be to lessen the uterine discharge, and promote the expulsion of the ovum." 110.

The recumbent position, saline aperients, cool temperature, and the absence of stimuli, constitute the chief means requisite on this occasion. To these may be added perhaps, in extreme cases, the local application of cold. The use of poisons, as digitalis and acetate of lead, is justly reprobated by the author as dangerous, and we may add totally inefficacious, in these cases. Besides the fatal effects which may be exercised on the ovum, while the maternal system is labouring with the sedative operation of these medicines; the cerebellum and medulla spinalis of the patient are exposed to the same paralyzing and often permanent lesion by the internal as by the external use of the latter remedy. In this, as in other species of hæmorrhage, the surgeon should not be guided in the application of his resources by the quantity of blood lost, but by the effect produced upon the individual constitution: he should not prescribe for the disease, but for the patient. The collapse, which a delicate person may experience from uterine hæmorrhage at this early period, has been sometimes fatal. The author adduces four cases of this description. To obviate such unpleasant results it is fortunate that we possess, when consulted in proper time, an expedient as powerful as it is simple: we mean the *vaginal plug*.

"This grand agent, though noticed by many of the earliest writers, was not applied at all conformably with the principles of science, until advocated by Leroux. It is evident, however, that the efficacy of the tampon (plug) is far from being duly estimated, even at the present day, notwithstanding all that Dewees, Burns, and other eminent modern practitioners have advanced in its favour. The uterus, from the closeness of its texture, and the small size of its cavity, will resist distention in the early months. But in the latter months, this is not the case; for, as its substance has then become very ductile and yielding, its cavity capacious, and its length greatly increased, accumulation will more readily take place; and since this is the objection which attaches to the plug, it may be asked, up to what period of gestation can it be applied without incurring the risk in question? I should say, certainly up to the beginning, or perhaps the end, of the fourth month. Its advantage, particularly about the third month, is very striking. The vessels will then have acquired a size sufficient to yield a copious effusion, and our other manual resources are at this time very limited. The rupture of the membranes is rarely justifiable before the sixth month. Before the fourth month, this measure is quite out of the question; but even were it otherwise, since all possibility of the continuance of gestation terminates with this operation, which does not necessarily attach to, although it very generally follows, the use of the plug, it is most fortunate that we have so effective an agent within our control. It may be laid down as an axiom, that the hæmorrhage which accompanies an early abortion, ought never to proceed to the direct destruction of life; for, since the uterus cannot be distended by effused blood until the fourth month, and as the tampon will certainly command the hæmorrhage, death from this cause will generally be prevented when the remedy is timely and properly applied. Notwithstanding this, fatal results are every now and then allowed to happen." 112.

"For the purpose of plugging the vagina, a soft sponge soaked in vinegar, solution of alum, or other styptic, is usually selected; but sponge, unless smeared with ointment, or steeped in oil, is not well adapted for the purpose; since, from its porosity, the blood is not completely coagulated, the liquid parts passing through its substance. I give the preference to lint, cotton-wool, or a soft handkerchief. The removal of the plug, of whatever substance composed, is facilitated if oiled before it is used. The T bandage may then be applied.

The plug is peculiarly serviceable in two conditions of the uterus—first, when the hæmorrhage is great, and the os uteri firm and unyielding; and secondly, when the flooding has so far depressed the system as to leave the uterus incapable of acting. The pressure of the plug excites the organ to contract. Fully to obtain its effect, it is material that the vagina be thoroughly filled with the substance we employ. If the plug only occupies the cavity partially, the blood may continue to escape, as I have frequently noticed, and thus defeat our intention. Several small pieces are preferable to a large single piece; the application is easier, the pressure greater, and the coagulation more readily affected. If, as occurred to me in one case, the urethra is so compressed by the plug as to cause obstruction of urine, we can remove the piece last introduced, without at all disturbing either the other pieces or the coagulum formed at the os internum. Under this treatment, the deciduous membrane may possibly not pass off entire, as under its ordinary expulsion, but escape in shreds. The discharge also becomes more offensive, until the whole contents of the uterus are cast off. But the expulsion of the ovum, when entire, is sometimes delayed several days even after the plug is withdrawn; although, under an active contraction, the ovum may be soon expelled, and the uterus recede a little in the pelvis, leaving it resting on the plug. Dr. Dewees authorizes the removal of the plug after twelve or fourteen hours. It should not, in my judgment, be removed under twenty-four hours. Anterior to this period, the constitutional powers will not have sufficiently recovered to bear a subsequent hæmorrhage, should the withdrawal of the plug be followed by it; but, after that time, Nature usually will be adequately recruited to bear a recurrence, (though the bleeding rarely recurs,) until the plug can be replaced. After twenty-four hours, moreover, the coagula surrounding the plug, from confinement, becomes (become) extremely offensive, and on this account requires (require) to be removed. In a case attended with a most alarming exhaustion, in which I employed the plug three weeks after delivery, the hæmorrhage soon recurred on its being withdrawn, and continued several hours, until a large piece of placenta was expelled. Though the uterus was too firmly contracted to admit of a renewed distention, this second hæmorrhage had very nearly proved fatal. When, therefore, the patient's state, at the expiration of twenty-four hours, is such as leads us to fear the consequences of a return of the hæmorrhage, it will be improper to remove the plug under forty-eight hours." 115.

Rupture of the membranes is not recommended before the sixth month. Should hæmorrhage after the fourth, expose the life of the patient to imminent danger, it may be proper to have recourse to the operation. At this immature period it will however be found a difficult affair, except in the hands of those well acquainted with the anatomical structure and axis of the cervix uteri. To such we need not point out the manner in which it may be accomplished. The object in this case being to lessen the volume of the uterus and the diameter of the bleeding vessels, we do not conceive it necessary to dilate the cervix uteri, as the rupture of the membranes, if that measure should be indispensable, may be effected without exposing the patient to such unnecessary pain and delay. We agree, however, with the author, whose sentiments and conduct seem to be influenced in every instance by humanity, that such a step should never be taken, except when maternal life is exposed to imminent danger. Circumstances may arise to render other assistance occasionally proper: as when the placenta has been partly protruded through the os uteri, and hæmorrhage is co-existing. Here a little aid may liberate the mass, and allow the matrix to contract. For this purpose instrumental relief has been advised by accoucheurs, of which the polypus-forceps, recommended by Gooch, is perhaps the most innocent. Having never found it necessary to use any auxiliary in addition to our own fingers or hand, we are of opinion that instrumental help may be generally avoided. The following case points out the advantage, which may be derived from the interference of art on these occasions.

"I have in my possession an entire ovum, which was brought away after death. In this case, after repeated hæmorrhages, the patient died; and, on opening the body, the ovum was found totally detached from the womb, and just at the point of descending into the vagina. A slight effort would have released it, and most probably have preserved a valuable life. In order to remove the ovum, it may be necessary to pass the entire hand into the vagina." 117.

After abortion and premature labour, the placenta is sometimes detained several days or weeks. This detention is liable to be followed by hæmorrhage, inflammation of the uterus and contiguous viscera, or irritative fever of a formidable nature. This occurrence is generally produced by a partial contraction of the uterine fibres near the cervix. The remedies, which the author proposes, consist of frictions, external pressure, cold, purges and the ergot of rye. He appears to entertain great solicitude about these cases, and relates an example in which delirium, convulsions and death, followed on the seventh day after premature delivery, the placenta being retained.

When the *lochial discharge* continues considerable during many weeks, the author states that injections into the uterine cavity are exceedingly useful. As he has omitted to mention the preparation he prefers for this purpose, we may observe that we have found a composition, consisting of sulphate of zinc and alum, each one drachm, dissolved in a pint of water, possess the greatest efficacy. As an auxiliary, the most important is the recumbent position, which will promote at the same time the proper application of the injection. The author also strongly recommends pills, consisting of sulphate of zinc and opium. In all increased secretions from the mucous membranes (and the inner coat of the uterus belongs to that class), we have seen good effects from the internal use of the former medicine. When combined with a bitter extract or the sulphate of quinine, as circumstances may require, there will be found no occasion to add opium; as we have never observed sickness to follow the exhibition of such a combination. Galls, kino, catechu and other astringents have also been employed in these cases; and certainly most decided benefit has resulted in our practice from kino in union with alum.

The following remark, published by Dr. Lee in the article "Abortion" in the "Cyclopædia of Practical Medicine," is thus criticised by Mr. Ingleby. We will insert the author's critique in his own words.

"By far the most frequent cause of abortion is in the product of conception itself; viz. in a diseased condition of the fœtus, or its involucra."—*Cyclop. of Pract. Med. p.* 10.

"The result of my examinations of the ovum cast off in abortion, both in its healthy and diseased state, is the converse of what is here stated. I have found the ovum healthy in all its parts, even in women who have aborted a number of times in succession. If the case were as Dr. Lee represents, mal-formations would cease to be an exception to the ordinary course of Nature, and treatment would rarely be of any avail."—*Ingleby, p.* 120.

The *hæmorrhages* occurring in the last three months of pregnancy the author divides into *accidental* and *unavoidable*. In the former the blood may proceed from the large vessels supplying the placenta, or from the smaller ones nourishing the decidua and connected with the membranes. When the effusion is sudden and copious, it will be found to proceed from a detachment of the placenta; on the contrary, when small in quantity and of a pale and more fluid nature, we may conclude that it arises from the decidua, and is not likely to impair gestation. Partial separation of the placenta may be owing to an excited state of the circulation, produced by stimulating diet or mental emotion; external injury, &c. The author adds also tenacity of the margin of the placenta as, in his opinion, an occasional cause. Manual examination is absolutely necessary in these cases, to ascertain the state of the os uteri and the nature of the presentation. The recumbent position, and the usual means of preventing a return of the flooding should be immediately and carefully pursued; and, should these fail, we must not allow valuable time to be wasted, till the vital powers are exhausted; but should decide at once upon the best active measure, which experience may suggest. Two means have been proposed to answer this purpose: one consists in rupturing the membranes in order to produce immediate uterine contraction; the other in proceeding at once to effect delivery by turning the child. Each plan has its advocates. We quite agree with the author in giving a decided preference to the former; being satisfied from extensive experience that it will be found by far the more successful one. Should such a step fail, which we scarcely believe it would, to arrest the hæmorrhage; especially if pains were taken to promote the discharge of the liquor amnii; we should have no difficulty in completing the entire evacuation of the uterus: as in that case such an atony in its fibrous structure would be found to exist, or would readily admit the ulterior remedy of turning. The author here very properly observes, that should an irrisistible obstacle to this proceeding be discovered in the rigid contraction of the uterus, that very circumstance must render such a measure needless; as a suppression of the hæmorrhage would almost invariably supervene. In the operation of turning, too, there is always some risk of rupturing the uterus, especially when its sides have become attenuated by long pressure or relaxed by repeated parturitions; not to mention the possibility of death taking place during the operation, an event, which has occasionally happened. The state of the os uteri, the constitution of the patient, and the effects of the hæmorrhage ought to be carefully ascertained. When the orifice of the uterus is thick and rigid, when the subject of the case is very delicate and liable to attacks of syncope, and her mind depressed by alarming presentiment of death, and when a state of collapse exists, or is impending, we feel confident that the issue will be more frequently successful by the gentle procedure; and, should the patient survive delivery, there will be much less danger of inflammatory action succeeding in the serous or interstitial membranes. When we have proof of an extensive separation of the placenta, and the state of the patient and os uteri will admit, we may find it expedient to proceed to delivery without delay; but as a general rule of practice, the other course is the more safe and prudent, and possesses the almost universal sanction of experience.

For the reason, which the author has stated before, the *plug*, in this advanced stage of pregnancy is an inappropriate and, in a relaxed condition of the uterus, a dangerous remedy.

In *rupturing the membranes* the author prefers the finger to any artificial contrivance; and, should the discharge of water be obstructed, he approves of the practice of raising the fœtal head. In every case, except one, in which this plan has been adopted to arrest uterine hæmorrhage, he has had the satisfaction of accomplishing his wish, and of witnessing the natural termination of the parturient process, without farther aid from art.

While engaged in our examination of the os uteri, we should be careful, if possible, to ascertain whether the placenta may have formed any connexion with it; as our practice of course must be adapted to that circumstance.

Instances of alarming hæmorrhage may happen, which cannot be restrained by the rupture of the membranes, and which may not admit of delay. In these cases we must proceed, in the most expeditious and prudent manner we can, to evacuate the uterus, with such mechanical assistance as our experience may suggest.

Much difference of opinion has prevailed respecting the dilatability or capacity of the uterus to contain more than its natural contents at any given stage of gestation. The author believes that it does possess this property; and the fact has been established beyond all doubt by the case of *internal uterine hæmorrhage*, published by Mr. J. M. Coley of Bridgnorth, in No. 332 of the Lancet, and by the post mortem examination of a patient of Mr. Saumarez, referred to by Mr. Coley in his remarks on that disease. This elastic property of the uterus seems to increase in proportion as gestation advances. Hence the danger of confiding in the employment of the plug in the last two or three months; since we find that, when the discharge of blood is obstructed by any natural impediment, the hæmorrhage proceeds internally with so much celerity, as speedily to destroy life.

The subject of *internal uterine hæmorrhage* is next explained, in reference to the small appearance of blood externally; and in alluding to that obscure species, in which no external discharge takes place, and which was formerly considered irremediable by art, the author thus proceeds:

"It cannot be denied that sudden deaths, occurring in advanced pregnancy, without any internal issue of blood, correspond not only with detachment of the placenta, but with laceration of the mass also; the blood being thus permitted to accumulate in a considerable quantity. Under the rupture of a central vessel, the placenta has been found so much detached, and forced inwards, from its corresponding uterine surface as to enclose at least two pounds of blood, and as the effusion is bounded by the edges of the mass, which still adheres, it cannot escape into the general uterine cavity." 136.

In illustration of this remark, the author refers to a short Essay on *Internal Uterine Hæmorrhage*, published by Mr. J. M. Coley in the Lancet, No. 332, in which is recorded an alarming case, that terminated successfully by the artificial rupture of the membranes, and the subsequent evacuation of the uterus. In this case the surgeon in attendance, being unable to discover the nature of so singular a disease, requested the opinion of Mr. Coley, who perceiving that the patient was labouring under that peculiar species of hæmorrhage, which he has denominated internal uterine, directed the membranes to be instantly ruptured. This operation was followed by immediate relief, and delivery was effected about two hours afterwards by the natural efforts, assisted by secale cornutum. The lady having been almost in articulo mortis during several hours, before Mr. Coley was consulted, he acted very prudently in taking the course which we have described. In such cases, the skin cold and clammy, and the pulse and senses imperceptible, the least violence, increase of pain or of hæmorrhage, would have proved instantly fatal; and repeated experience has convinced us, that at such a critical moment, manual delivery is almost certainly destructive.

This peculiar variety of hæmorrhage has also been noticed by Burns, Baudeloque, Albinus, Saumarez and Blundel.

*Rupture of the placenta* sometimes happens. A case is related in the North of England Medical and Surgical Journal, No. 4, p. 446; and a similar accident was noticed by the author in a cat, which had died from internal hæmorrhage. After describing the symptoms denoting internal uterine hæmorrhage, he adds—

"We shall be quite justified in passing the hand in utero (provided it can be done without great violence) and accomplishing delivery." 138.

Before we adopt this course, the state of the os uteri and of the patient should be ascertained. When the former is only partially dilated, thick and rigid, and the patient in danger of expiring every moment from a long-continued collapse; the only course, which can afford her a chance of recovery, will be to adopt Mr. Coley's proceeding of rupturing the membranes, which should be done in the most gentle manner. When the os uteri is found dilated or very thin, and the patient labouring under only the ordinary effects of a common flooding, without any of that characteristic appearance of distress, death-like coldness and clamminess of the skin, and absence of pulse, accompanying alarming collapse, the surgeon may proceed at once to effect manual delivery with great prospect of success. It should be observed that this accidental separation or rupture of the placenta is liable to happen, unconnected with the process of parturition; when the plan of rupturing the membranes will possess still greater advantages to recommend it.

The *unavoidable hæmorrhage* is that, which proceeds from a separation of the placenta, after it has been attached to the inferior part of the uterus. When flooding arises from this cause, it must necessarily increase with the dilatation of the cervix and os uteri, which commences about the fifth month. The author states that it has been witnessed as early as the third, but it is seldom met with, until some period after the fifth month. These hæmorrhages, if left to Nature, would, with a few exceptions, be fatal. The placenta and fœtus have been expelled together, and the former first and the latter afterwards with safety. These however are rare occurrences. The placenta may be adherent wholly or partially over the uterine orifice. The exact nature of the case should be ascertained by careful examination. After attempting relief by decubitus on a hair-mattress, by cool regimen, saline aperients, injections, and the application of cold; should the discharge continue to increase, or return so suddenly and frequently as to produce alarming effects, the author recommends delivery to be effected with the following precautions, which we are glad to find correspond with those we gave, when speaking of internal uterine hæmorrhage.

"A forcible entrance into the uterus, whilst its orifice is rigid, is never to be justified."

"If the os internum be tolerably soft, and dilated to the size of half-a-crown, or, what is of far more importance, soft, thin, and dilatable, sufficient to admit the finger easily, the operation may be attempted. On gently passing the ends of the fingers in succession, and in a conical form, we quickly ascertain the degree of resistance. By pausing a little, if needful, relaxation may follow; if not, the hand must be withdrawn, and the attempt renewed at the proper time." 146.

As soon as the requisite relaxation of the os uteri has taken place, we should proceed to deliver, as any unnecessary delay might be dangerous or fatal. The author's ample experience and that of his friends have established the decided advantage of early delivery in these cases; and the welfare of the infant and parent is alike dependant on the promptitude and skill of the accoucheur. The author here exhorts us to practice great perseverance in our efforts to restore suspended animation in the child after birth; and adds that he succeeded in one instance after 50, and in another 55 minutes had elapsed.

When delivery cannot be safely attempted, either on account of extreme collapse or an unfavourable condition of the os uteri, the use of the plug may occur. The author states forcible objections to it in the state last mentioned, particularly the impossibility of following every fresh laceration of the placenta with such a remedy. Being desirous of obtaining the sentiments of eminent accoucheurs on this subject, he has been favoured with those of Sir C. M. Clarke and Dr. Blundel, with which we will present our readers.

"Dr. Clarke, who does not seem to be apprehensive of its occasioning an internal hæmorrhage, is, notwithstanding, opposed to the principle of using the plug when any thing remains in the uterus to be brought away; he regards the practice as in itself unscientific, a half and uncertain measure, when decision and action are indispensable, and would seek to procure contraction by the only certain means. This eminent man, who always delivers as early as possible, never lost a patient in this presentation. Dr. Blundel, on the other hand, averse, as he has often declared himself to be, to officious midwifery, would not hesitate to plug either in general or partial placental presentation, 'provided (as he observes) the os uteri were rigid, and the gushing or draining seemed to require the remedy,' doubtless from the impression the loss shall have made on the system.

"Repeated deaths from placentar presentation prove that the unaided powers cannot be depended upon in all cases, whilst we are waiting for relaxation." 152.

When dangerous exhaustion exists, the author does not hesitate to pronounce the plug a valuable auxiliary till such reaction takes place, as may afford hope of success from the employment of more decided measures.

"Whilst flooding continues, the practitioner has but one duty to perform, viz. to deliver; but, when coldness of the skin, a pulse scarcely perceptible, (associated perhaps with vomiting,) and a countenance denoting excessive exhaustion, supervene upon an hæmorrhage that has temporarily ceased, a mere draining going on, such a moment is ill adapted for turning the child. There is an axiom in midwifery, that no woman should be suffered to die undelivered. I assent to this as a general rule; at the same time, its rigid enforcement during a state of collapse fairly admits of question—the mere bodily disturbance has too often proved fatal." 153.

Such a state as this is, he thinks, peculiarly adapted for transfusion.

"Rather than deliver under collapse, we ought to occupy ourselves in administering stimuli and cordials, promoting animal heat, perhaps performing transfusion, carefully watching the effects of reaction, and holding ourselves in readiness to deliver on the recurrence of bleeding, or, if the tampon be employed, as early as the patient's strength will allow." 153.

The authority of Dr. Blundel is again brought forward in recommendation of plugging in this exhausted condition; and in farther proof of its good effects, a case is appended, that came under the care of Mr. Grainger of Birmingham, who, finding his patient moribund, applied the plug, and waited two days, before he ventured on delivery.

The rupture of the membranes, when a considerable part of the placenta is fixed on the os uteri, is inadmissible near the full period of gestation. Any time before the fifth month it may be had recourse to, as at that early period the hand cannot be urged into the uterus, without using improper force. When it is found necessary to introduce the hand in the advanced stages, for the purpose of turning, the author prefers passing it between the placenta and uterus, rather than through the substance of the former, as has been recommended by some writers; but, when imminent danger to the mother is at hand, the placenta should be pierced without delay. The state of the patient and of the hæmorrhage should determine the propriety of expediting the extraction of the child and placenta, after the feet have been brought down.

The consequences of large and repeated losses of blood, especially when accompanied with temporary suspension of the functions of the heart and brain, are often most severe and distressing; and, after harassing the patient during several months or years, they sometimes terminate in organic disease in one of these important organs, particularly the former. After collapse of long continuance, reaction sooner or later succeeds, and is often associated with sub-acute inflammation of a most tedious or indomitable character. Various derangements of tiresome duration in the organs of sense, particularly that of hearing, follow; and, should the patient finally recover, she remains long unfit for any active duties. All stimuli should be abstracted, as soon as re-action begins; and this should be modified by suitable means, care being taken in the employment of aperients not to induce a diarrhœa, which may speedily prove fatal. With the author's remarks we will close this part of the subject.

"When the gushing or draining has continued a considerable period, the stomach being unable to supply sufficient nourishment to counteract the exhaustion, the evils peculiar to large losses of blood frequently ensue. Death has sometimes occurred at a comparatively remote period after delivery; in some instances, under an impaired state of the system generally, accompanied with dropsical accumulations; in others, from organic changes, in the heart and brain especially, the consequence of previous loss of blood." 162.

*Hæmorrhage occurring during labour* generally arises from a detachment of the placenta. The author seems inclined to believe this separation may be attributed to the use of ergot of rye. When interference is necessary, delivery must be hastened in the most practicable manner, either by turning or the forceps.

With respect to *flooding connected with plurality of children,* and which is generally occasioned by a busy interference, the author finds the most successful practice is to leave the expulsion of the second or third child to the natural efforts, which he has observed usually commence about six or eight hours after the birth of the first. By observing this rule he has been uniformly successful, and his patients have escaped hæmorrhage. Those, who advocate the plan of rupturing the membranes and delivering the second child within a given time, will find themselves frequently perplexed with an hour-glass, or other partial contraction of the uterus and most alarming hæmorrhage. To this mal-practice the intelligent author ascribes some of the most serious fluxes of blood, which he has witnessed. Were he disposed to fix a precise time, as a safe interval to elapse between the birth of the first child and the re-commencement of uterine action, he asserts that twelve hours would by no means be too long. In 85 cases occurring at the Birmingham Dispensary in ten years, the pains were not renewed on an average in less than twelve hours, excepting those cases, in which the second speedily followed the first birth. In a few instances, 24 to 30 hours elapsed. In the absence of pain the horizontal position, perfect rest and tranquillity should be enjoyed; and, when the patient has recovered, frictions, a firm bandage and the ergot of rye may be employed, and the os uteri stimulated with the fingers. Should some slight action commence, the delivery may then be facilitated by rupturing the membranes. The presenting part should be discovered at the same time, and the child turned, or the rest of the process left to Nature, as circumstances may require. Should hæmorrhage arise between the expulsion of the first and second child, delay will be unjustifiable.

The rules for the *management of the placenta* have been subject to great extremes. Dr. Hunter's practice of leaving the affair altogether to Nature, was found to be productive of most unfortunate results; and the author mentions a case of recent occurrence in genteel life, which terminated fatally in consequence of the placenta being allowed to remain in the uterus, until the fourth day. On the other hand a hasty or premature extraction of the secundines is liable to produce laceration of them, or prolapsus or inversion of the uterus, and will almost to a certainty occasion most alarming hæmorrhage. Two cases of inversion from this cause have come under the author's notice. One of them was succeeded by fatal hæmorrhage in three hours, and the other was irremediable, the patient being still alive.

When violent flooding takes place, no time must be lost in passing the hand into the uterus, grasping the placenta and exciting the former to contract, so that the hand and its contents may be expelled together. In the absence of flooding the author agrees with Dr. Joseph Clarke, that two hours should be allowed to elapse after the birth of the child before any attempts should be made to remove the after-birth. To effect this, particular directions are given to meet every exigency. In twin-cases a longer interval than two hours should be permitted. *Retention of the placenta,* according to the author's experience, may arise from torpor, or irregular spasmodic action of the uterus, or from morbid adhesion. It is generally provoked by impatience or unskilfulness on the part of the accoucheur. We sometimes find a quantity of blood confined in the uterus by coagula, or by the placenta lying loose at the orifice. The latter obstruction should be removed without delay; and when there is absolute want of uterine contraction, and the placenta is partly adherent, we are directed by the author to apply frictions over the abdomen in the region of the fundus uteri, and cold to the hypogastrium, and to administer ergot of rye; and, should these fail, to pass the hand into the uterus and to inject cold water into its cavity. When the constitutional exhaustion is extreme, and only a small part of the placenta is detached, the author says that the bleeding spot may be discovered and compressed with the hand in the uterus, until the circulation is restored and contraction begins; when the hand and placenta will be expelled together. This mode of suppressing uterine hæmorrhage was, we believe, first published by Dr. Gooch. His plan consisted in introducing the left hand, closed, within the uterus and applying the right, expanded, to the outside of the abdomen; and between the hands he compressed the bleeding vessels.

The spasmodic retention is denoted in extreme cases by a hardness in one part of the uterus of various size and shape, felt externally, while the other portions continue relaxed. It is generally attended with pain, and, when affecting the middle of the organ only, is called from its figure the hour-glass contraction. Some futile attempts have lately been made to disprove the existence of this species of constriction. Any one, who, in attempting to introduce his hand for the purpose of reaching the incarcerated placenta, has really felt this powerful, spasmodic stricture in the uterine fibres, could not possibly be mistaken. We have indeed met with it nearly at the fundus, certainly above the middle, especially in those cases, in which a part of the placenta has been strangled by the contraction. In support of this decided conviction of ours we are happy in being able to add the author's unshaken and respectable opinion.

"To the young practitioner, I allow, deception on this point is not unlikely to arise; for the inferior part of the uterus, being in a flaccid and dilated state, appears to be in direct continuity with the vaginal canal." 193.

Five cases are introduced, which prove satisfactorily the nature and existence of the disease. The fourth is so interesting and instructive, particularly in pointing out the absolute necessity of removing the obstruction by manual aid, that we cannot resist presenting it to our readers in the author's words.

"I was called twenty-six hours after the birth of the child to a woman with retained placenta, the funis having been disrupted at its point of insertion. The os uteri flaccid, and nearly the circumference of a dollar in size, could be as plainly discovered by the finger, as in the first stage of labour, and a portion of placenta was felt detached immediately above it. Although I succeeded in passing the os internum, it firmly embraced my hand. The detached portion of the placenta could be traced through a firm stricture in the centre of the organ, which with difficulty permitted my fore-finger to enter, and above which the placenta was firmly adherent. The day previous to this, the practitioner in attendance had made several attempts to pass the stricture. This gentleman assured me that no part of the placenta could at that time be felt, the stricture closely encircling the funis, which then gave way. When I saw the patient the following day, the spasm must therefore have become sufficiently relaxed to allow a piece of placenta to pass by it, which it embraced very firmly. Opium and castor-oil had been largely employed. The extreme turbulence of the patient compelled me to desist; she declared she would rather die than submit to the dilatation of the stricture. I was again called to the patient five days afterwards. She had bearing-down pains truly distressing, and as forcible as though the child was passing the os internum—the breathing was laborious, the pulse rapid, the fœtor intolerable, the danger extreme. The hand was again passed through the os uteri, but not through the second stricture, but a large portion of the placenta was now withdrawn through it. Death occurred on the thirteenth day. On examining post mostem, the body was uniformly healthy; a piece of placenta, about the size of a small egg, was attached to the fundus very firmly, and could not be separated either by the finger or maceration." 197.

In whatever part of the uterus the contraction may exist, it is our duty to remove it by a gradual dilatation with the hand, which, having grasped the imprisoned placenta and excited the fundus into action, should be entirely withdrawn. As an auxiliary, the author mentions opium among other remedies. When the pain is extremely violent and the spasm unusually obstinate, temporary benefit may probably be derived from it. Having however invariably found the pain to subside, as soon as the manual operation has been concluded, we have had no need of its assistance.

The *adherence of the placenta,* which may be general or partial, is a most serious circumstance. As soon as it has been ascertained, the author directs the hand to be introduced into the uterus, and the inner surface of the latter to be stimulated by moving and gently pressing the knuckles against it. Should great depression and hæmorrhage attend, the latter must be arrested with the two hands in the manner before described, until the vital powers are renovated. Then the surgeon must proceed to peel off the placenta, beginning at any point of its edge most easy of access. Having succeeded in entirely separating the adherent surfaces, the placenta must be held fast in the hand, until uterine contraction has been elicited and all the extraneous contents of the matrix expelled.

A partial adhesion of the placenta, alluded to by Dr. Ramsbotham, having a loose portion hanging over the os uteri or in the vagina, has often occurred in the author's practice. In attempting to remove the detached part, the placenta may be torn and the adherent portion left behind, to the imminent danger of the patient. When common expedients fail, the hand must be introduced. On the unguarded directions of Dr. Gooch, who recommends the placenta to be pulled with considerable force, the author comments with just severity, and subjoins a fatal case confirming the propriety of his censure. Whenever a part of the placenta is left adhering to the uterus, it should be removed immediately after delivery: for should this proceeding be delayed only a few hours, it will be found very difficult or hazardous to attempt the introduction of the hand for that purpose. The mass left behind is usually discharged afterwards in a state of decomposition. The period, when this may take place, is very indefinite: it generally happens in the course of four or five days. In one case the author states, that the adherent portion was not cast off in less than 21 days; and Ramsbotham relates another, after a premature labour, in which at the end of a month it was expelled in a healthy state, having undergone no apparent alteration. It has been supposed by Dr. Neagle that sometimes absorption of the dead mass takes place; and a friend of the author informed him that half of the placenta, in an instance under his own observation, being detained, was never afterwards found to pass away. The author mentions another result of disruption of the afterbirth, which, he believes, has not attracted the distinct notice of others. This peculiarity consists in such an intimate connexion between one circumscribed portion of the placenta and uterus, as does not admit any obvious line of demarcation between them. Hence he says—

"Instead of the retained portion being cast off by the progressive contractions of the womb, the connexion may be sufficiently close to resist these efforts of Nature; the organ remaining bulky, and the vessels, supplying the extraneous body, unusually large. If the hæmorrhage is inconsiderable, and the constitutional energies unimpaired, the mass, by acquiring an increased degree of organization, presents a florid hue, not unlike a fungus (fungous) growth, in place of the black and offensive structure which characterizes a disrupted state of the placenta. Moreover, the retained portion may become so far identified with the lining membrane of the uterus as to render a distinct and perfect disunion impracticable. Unless the mass be completely walled in, vessels will be exposed; and though decomposition may not take place, hæmorrhage will necessarily arise; and as the uterus cannot be perfectly contracted, the cessation of the hæmorrhage must entirely depend upon the formation of coagula within the vessels. On the clots being displaced, the effusion will be renewed from time to time; but so far from occurring immediately after delivery, several days may elapse before it appears. The dissections of women who have perished from hæmorrhages at different periods after delivery, fully establish my assertions. In one instance of this kind, hæmorrhage began on the third day after delivery, and, with the exception of a few short intermissions, continued during a period of five weeks, when it terminated in death. On inspecting the body, a tumour of rather florid colour and the size of the largest walnut, was found firmly adherent to the sides of the fundus uteri at its highest part; the lining membrane covered the greater portion of the mass, though not its centre, which was ragged, and vessels could be traced opening upon it." 210.

The consequences of retained portions of the placenta are often an hæmorrhage, irritative fever and inflammation, and suppuration of the uterine lymphatics and veins. The hæmorrhage is occasioned by repeated exposure of the blood-vessels and may terminate fatally. To obviate this result, we should, if possible, remove any adherent portions within reach of the fingers. The author very properly questions the propriety of using the plug in this case, as, the hæmorrhage being generally attended with a putrid discharge, it might endanger the life of the patient to confine the latter within the uterus. Should the flooding consist purely of blood, and the uterus be sufficiently contracted, there could exist no doubt about the safety of the practice.

In irritative fever, arising from detained placenta in a state of decomposition, the following state more or less prevails.

"The sensorium is early affected, as denoted by pain in the head and wandering of mind—an anxious, pallid, or sallow countenance, vomiting, rigors, thirst, heat and dryness of skin, great rapidity and feebleness of pulse, flaccidity of the breasts (for milk is rarely secreted), tremors, which, when the uterine pain is severe, pass into violent convulsions, and sometimes augmented sensibility over the hypogastrium. The bowels are at the onset confined, but diarrhœa frequently prevails in the course of the complaint. As the disease advances, there is delirium or stupor, slight but uninterrupted convulsions of one or both arms, and sometimes the face, a dry and brown tongue, lips and teeth covered with sordes, a puffy and tumefied state of the abdomen, fluttering of the pulse, and involuntary evacuations." 213.

Irritative fever, when fatal, generally terminates from the eighth to the twelfth day; sometimes life is protracted till the third week. In almost all his cases the author has observed—

"No disease or vestige of inflammation on dissection; on the contrary the body seemed drained of its blood. The state of the system differs little, except in aggravation and the presence of a putrifying mass, from that described by Dr. M. Hall, in his Researches, as the result of losses of blood; and the necessity of a stimulating practice here is now universally admitted." 221.

To dilute the morbific property of the putrid contents of the uterus, from the absorption of which it is supposed that this species of fever arises, the author proposes the injection of tepid infusion of chamomile with liq. calcis vel sodæ oxymurias; and, after the offensive odour has subsided, he recommends cold solutions of alum to be thrown into the uterus, by introducing the point of the injecting instrument within the os uteri. An elastic gum-bottle, or the improved vagina-syringe will answer the purpose. The linen should be often changed, and the apartment ventilated. The constitutional treatment at first may consist of mild, saline aperients, followed by the citrate of potash; and symptoms may require sulphate of quinine, subcarbonate of ammonia, aromatics, &c. To allay restlessness the author advises acetate of morphia, and solid opium to relieve vomiting or purging. A liniment, composed of ol. terebinth. and lin. sap. c. is much extolled by him, as an external application to the uterine region.

*Inflammation from detained placenta* happens mostly on the second or third day after delivery. The author asserts that the lymphatic has appeared to him more immediately affected than the venous system. In one case, though attended with phlegmasia dolens, no inflammation in the veins was discovered. The symptoms denoting inflammation in the lymphatics and veins of the uterus have unfortunately been omitted by the author. Notwithstanding he might have considered it a digression from the subject of hæmorrhage, it would have rendered this portion of his treatise more complete and valuable, on account of the extensive opportunities, which he must have enjoyed for accumulating practical information on the disease. In speaking of the treatment of this species of inflammation, he observes that general bleeding is rarely admissible.

"Should there be much pain in the back, eight or ten ounces of blood may be abstracted by cupping the loins; otherwise the application of leeches both within the vulva and over the hypogastrium, is much to be referred. The abdomen may be fomented, and a large and soft linseed poultice, (if its weight can be borne), afterwards applied. Relief will also be obtained by injecting into the uterus a decoction of poppy heads, or a weak solution of the extract of belladonna in water. The French practitioners have eulogized the action of mercury in inflammation affecting the uterine structures. I have seen its full specific effects obtained within a short time, without any relief to the symptoms." 217.

Nine fatal cases, interspersed with remarks, and some necrotomical observations conclude this part of the work.

The author, having described the different states, in which the uterus may be found after delivery, proceeds to notice the *hæmorrhage with a firm contraction of the uterus*, first pointed out by the late Dr. Gooch, and attributed to an extraordinary force and frequency of circulation, which does not permit the orifices of the vessels to contract. The absurd explanation of this singular species of hæmorrhage, attempted by a Mr. Roberton, is ably exposed by the author, who has annexed the following case to his remarks.

"I had an opportunity of noticing this species of hæmorrhage in the person of a young lady of naturally spare habit of body. During her first pregnancy, she attained a degree of corpulency and robust health very remarkable, when it is considered that from the activity of the absorbent system attenuation is for some months a characteristic symptom of gestation. After the disengagement of the placenta, although the uterus was reduced to as small and tonic a state of contraction as it ever attains at this period, the hæmorrhage was for some time exceedingly profuse, and was not restrained by pressure. The pulse was unusually strong and full when the hæmorrhage commenced. In a case like this, the institution of physical treatment would be highly proper, particularly towards the close of gestation; but during labour the hæmorrhagic tendency is to be subdued by observing a low temperature, cooling diet, perfect repose, and venesection if needful. This form of hæmorrhage occurred in the practice of Mr.

Porter, of this place, after the delivery of twins. When the hæmorrhage commenced, the pulse was full and strong, and the uterus in a state of high tonic contraction. Within two hours the vital energies became so dangerously depressed, as to lead us to contemplate transfusion. The patient was talking most incoherently, the pulse was apparently absent in the left arm for nearly two hours, and was barely perceptible even after the circulation in the right arm had been materially restored. The uterus nevertheless remained in a state of firm contraction. Dr. Gooch advises the left hand to be introduced into the uterus *closed*, and the right to be applied open on the outside of the abdomen, so as to compress between the two the bleeding surface." 229.

That variety of *internal or concealed uterine hæmorrhage*, which occurs after the expulsion or extraction of the placenta, generally takes place within the first hour.

"The patient becomes pale, complains of being very faint, feels sick, perhaps vomits,—the heart beats feebly and rapidly,—the exhaustion progressively increases. The practitioner no longer finds the contracted uterus in hypogastrio; the abdomen is occupied by a soft and large tumour, occasioned by the uterus being distended with blood. There is little or no external discharge, the napkin being scarcely soiled, and the os uteri will be found more or less plugged up with a coagulum." 232.

To these symptoms death very often rapidly succeeds. The author here enumerates the severe impressions made upon the brain, the heart, the stomach, the lungs, and capillary circulation, by the sudden extravasation of the vital fluid within the uterine cavity; and adds some interesting observations, deserving the most serious attention, and evincing the importance of ascertaining the nature and extent of the *effects* resulting from the sanguineous effusion in every individual constitution, together with the influence previously excited upon it by moral and physical agencies.

The plan of treatment suggested by the author consists in the exhibition of the ergot of rye, and, when exhaustion is extreme, of opium; frictions and manipulations on the region of the uterus, with the view of provoking its contraction; the application of cold air; and the elevation of the hips, the head being at the same time depressed. When the blood, contained in the uterus, is chiefly liquid, it may be forced out by external pressure, which will be followed by speedy contraction. Coagula however of enormous size often form, which cannot be dismissed by such an easy process. In this case, when alarming symptoms exist, we have repeatedly adopted with complete success the plan pursued by the author, which is the only effectual proceeding in such an emergency, namely the introduction of the hand into the uterus. Objections have been started to this measure from an imaginary fear of rupturing the relaxed and extended organ. In a long note on this subject, the author satisfactorily proves, from a comparative examination of numerous cases, that rupture commonly happens during a forcible contraction, and seldom during a collapsed condition of the uterus. In addition to the recommendation and experience of the author, we may state that this practice has received the sanction of the most respectable writers and practitioners of the present day. In the introduction of the hand great care is required, and, while danger is imminent, in addition to the pressure with the two hands over the placental region, as practised by Dr. Gooch, Mr. Ingleby proposes the compression of the large abdominal vessels at the same time. He places no confidence in the introduction of mineral or vegetable acids, and considers all similar remedies, when compared with the hand, as trifling and unsafe, excepting the injection of cold water, which he confesses he has observed to produce contraction, when the hand has failed in consequence of the bulky and flaccid state of the uterus. With respect to the plug in these cases, he considers it inadmissible in less than a few days after delivery, at which time he supposes the uterus may have lost its disposition or capacity for distention. This rule may be liable to a few exceptions, which none but the most scientific and experienced can discriminate. The only safe circumstances, under which plugging can be resorted to, are a comparatively contracted state of the uterus; the lapse of some hours after delivery; the hæmorrhage being dangerous; and the surgeon in constant attendance. At the same time it should be observed that death may take place from hæmorrhage at the distance of a fortnight after delivery.

Another resource in this emergency remains to be noticed, *i. e.* that of interrupting the current of blood through the abdominal aorta, first proposed by Plouquet. Experience, as far as it has extended, appears to confirm the good opinion originally entertained of this valuable remedy. There are two modes by which this object has been attained: one by the application of pressure externally on the abdominal parietes above the uterus; the other by introducing the hand within that organ and compressing the vessel through it. The first species of pressure, it is obvious, can only be adopted, when the patient is of spare habit of body; the other is applicable in persons of any dimensions. It is only with the former proceeding that the author has had any experience. This however is highly satisfactory, as will be seen from the following cases, in one of which this mode of treatment was adopted under his own immediate inspection. The first occurred in the practice of Mr. Blount, a respectable and experienced surgeon in Birmingham; the second in that of the same gentleman in conjunction with the author.

"On the termination of a labour Mrs. W. was to all appearance comfortable. In about half an hour she complained of being very faint; her countenance was very pale, and anxious. There had been little or no external hæmorrhage, and on placing my hand over the abdomen I could not distinguish any thing resembling the uterus. By friction and compression some discharge was forced away, but no beneficial effect resulted; the hand was therefore passed into the uterus, and a mass of coagula which distended its cavity cleared away. By this means, and by gently irritating its inner surface, contraction was promptly effected. Though the discharge had now considerably abated, the exhaustion was very great, the pulse being exceedingly feeble and frequent, and the patient appearing sinking. I now placed my hand on the abdomen just above the uterus, and, from the spareness of habit, easily distinguished the pulsation of the aorta, and made pressure so as to interrupt its current of blood. This was continued with increasing advantage for half an hour, and as the patient was much revived, I then withdrew my hand. Upon the withdrawal of the hand the sinking immediately returned, attended with giddiness in the head and a slight return of the hæmorrhage. Pressure was again made for the space of two hours with the same good effects. Twice during this period I allowed the blood to pass the lower extremities, but with the same unfavourable results as before. A modified and less effective pressure was continued for four hours longer, before it could be entirely dispensed with." 250.

"I recently assisted Mr. Blount in the treatment of a case resembling the foregoing. Whilst pressure was exerted over the aorta, the uterine contractions were provoked by two fingers passed through the os uteri. Whenever the pressure over the vessel was withdrawn, there was an almost instantaneous return of hæmorrhage and syncope, and some hours elapsed before the patient's safety was secure." 250.

Compression of the aorta by the hand introduced within the uterus is an operation, which seems to be principally adapted for a short period after delivery, while the os uteri remains sufficiently patent to admit the hand without violence.

It has been had recourse to with success by Mr. James of Westbromwich,* and three times by Dr. Eichelberger.†

The application of pressure by means of a large, circular plate, proposed by Mr. Mills, next attracts the notice of the author, who apprehends it will disappoint the expectations of its inventor. When external pressure is requisite, we have always found the hands the very best means for effecting it; being possessed of sensation, they afford us a knowledge of the state of the uterine fibres, and admit of being adapted so as almost to surround the organ, which we wish to compress; while they enable us to accommodate the degree and direction of the force, as the varying circumstances may demand. On the contrary, while the surgeon is flattering himself that his mechanical force is proceeding in due order, he may unexpectedly find that, its operation being limited to one point, namely in a direction towards the spine, the uterus may be forced out laterally by an insidious but fatal torrent.

The author here directs our attention again to the awful responsibility we should feel in the treatment of these appalling hæmorrhages, and urges us to the patient application of every possible expedient to resuscitate the suspended circulation and excitability. Here the least manual violence would be death. Instead therefore of disturbing the conservative process of coagulation, when the least unnecessary commotion may lacerate the thread of life, he recommends our attention to be occupied in directing the administration of external stimuli, and aiding their operation by diligent perseverance, and by inspiring hopes in the desponding assistants. In these perilous cases, the absence of pulse may continue ten or twelve hours, before re-action begins; and, when recovery appears to be proceeding, we must be careful to exercise the greatest circumspection both with respect to treatment and prognosis. The energy of the heart may not be restored by its temporary slumber; it may answer for a while to the spur of medicine, or be urged forward by labouring instinct; it may revive and flutter and then be still, perhaps for ever! While the power of deglutition continues, one of the most safe and potent stimuli is the subcarbonate of ammonia.

At distant periods after delivery, the author is disposed to recommend small doses of acetate of lead. Fully sensible of the nature of the ulterior effects of this dangerous medicine in some constitutions, he accompanies his directions for its use with very prudent cautions.

After severe hæmorrhages of this kind the recumbent position should be extended much beyond the period allowed on ordinary occasions. This measure may be assisted by gentle tonics and by astringent injections. The author extols the sulphate of zinc as an internal medicine in combination with opium—one or two grains of the former with a quarter or half a grain of the latter. Should opium be objectionable, ten grains of any bitter extract, as we have noticed before, will answer the same purpose. The injections may be composed of alum and sulphate of zinc. In addition to the invigorating remedies some prudent directions follow respecting the choice and exhibition of restorative diet. In one case the author found it necessary to administer food by means of the stomach-pump. A much more simple contrivance for this purpose consists of an elastic tube and bottle. To these directions succeed seven cases of uterine hæmorrhage, possessing considerable interest, which we regret our limits will not permit us to transcribe. The last, in which the operation of transfusion was performed, reflects infinite credit on the skill and promptitude of the author and of Mr. Wood, the celebrated operating and consulting surgeon at Birmingham. For the detail of this as well as the other cases, we must refer our readers to the work itself.

* Lancet, No. 235.

† Siebold's Journ. Geburtshuelfe, 1829.

That condition of the sanguiferous system, in which a deficiency of fibrin and red globules has been induced, is next described, under the title *anœmia*, which signifies simply want of blood. This state, so familiarly known by the profession, and by accoucheurs in particular, need not here be particularized. The treatment, both medical and dietetical, pointed out by the author, is highly judicious.

The work concludes with some remarks on *transfusion of blood*. The manner of performing the operation is extracted at length from Mr. Waller's Elements of Midwifery, to which we beg to refer our readers, having left ourselves no space for further extracts. The author, whose good opinion of the operation, as stated before, has been derived from personal observation, thinks that every practitioner of midwifery should be in possession of the transfusion-syringe.

The length, to which we have extended our analysis of the present article, leaves but little space for general observations. As a specimen of zeal, industry and erudition, is is highly creditable to the provincial school of which the able author is one of the professors. To all those engaged in the study and practice of midwifery we can recommend the work as the only complete and comprehensive book on the extensive and important subject of uterine hæmorrhage; and every student and practitioner, who is ambitious to attain and display a masterly knowledge of this department, should not only peruse Mr. Ingleby's Treatise with attention, but deposit it on his shelf as an indispensable work of reference.

